# A Case of Multiple Sclerosis—Like Relapsing Remitting Encephalomyelitis Following Allogeneic Hematopoietic Stem Cell Transplantation and a Review of the Published Literature

**DOI:** 10.3389/fimmu.2020.00668

**Published:** 2020-05-05

**Authors:** Joyutpal Das, Atta Gill, Christine Lo, Natalie Chan-Lam, Siân Price, Stephen B. Wharton, Helen Jessop, Basil Sharrack, John A. Snowden

**Affiliations:** ^1^Department of Neurology, Salford Royal NHS Foundation Trust, Manchester, United Kingdom; ^2^Department of Neurology, Royal Hallamshire Hospital, Teaching Hospitals NHS Foundation Trust, Sheffield, United Kingdom; ^3^Department of Haematology, Royal Hallamshire Hospital, Teaching Hospitals NHS Foundation Trust, Sheffield, United Kingdom; ^4^Sheffield Institute of Translational Neuroscience, University of Sheffield, Sheffield, United Kingdom; ^5^Department of Histopathology, Royal Hallamshire Hospital, Teaching Hospitals NHS Foundation Trust, Sheffield, United Kingdom

**Keywords:** multiple sclerosis, allogeneic hematopoietic stem cell transplantation, “domino” autologous hematopoietic stem cell transplantation, graft versus host disease, multifocal leukoencephalopathy

## Abstract

Complications involving the central nervous system (CNS) occur in 9–14% of patients following allogeneic hematopoietic stem cell transplantation (HSCT), including stroke-like episodes, demyelination, encephalitis, and nonspecific neurological symptoms. Here we report a case of multiple sclerosis (MS) like relapsing remitting encephalomyelitis following allogeneic HSCT, which did not respond to disease modifying therapies (DMTs) and “domino” autologous HSCT. A 53-year-old male was treated with allogeneic HSCT for lymphoid blast transformation of chronic myeloid leukemia. Ten months later he presented with confusion, slurred speech, left sided facial weakness and ataxia. A magnetic resonance imaging brain scan showed multiple enhancing tumefactive lesions. Neuromyelitis optica (NMO) and myelin oligodendrocyte glycoprotein (MOG) antibodies were negative. After extensive investigations for infections, autoimmune disorders and recurrence of malignancy, he underwent brain biopsy, which showed a macrophage rich lesion with severe myelin loss but axonal preservation indicating a demyelinating pathology. Although his symptoms improved with corticosteroids, he relapsed five months later. In the absence of any systemic features suggesting graft versus host disease (GvHD), his presentation was thought to be compatible with MS. The illness followed an aggressive course that did not respond to glatiramer acetate and natalizumab. He was therefore treated with “domino” autologous HSCT, which also failed to induce long-term remission. Despite further treatment with ocrelizumab, he died of progressive disease. An autopsy limited to the examination of brain revealed multifocal destructive leukoencephalopathy with severe myelin and axonal loss. Immunohistochemistry showed macrophage located in the perivascular area, with no T or B lymphocytes. The appearance was unusual and not typical for chronic MS plaques. Reported cases of CNS demyelination following allogeneic HSCT are very limited in the literature, especially in relation to histopathological examination. Although the clinical disease course of our patient following allogeneic HSCT resembled an “MS-like” relapsing remitting encephalomyelitis, the autopsy examination did not show any evidence of active inflammation. The impact of DMTs and HSCT on the histological appearance of “MS-like” CNS pathologies is unknown. Therefore, reporting this and similar cases will improve our awareness and understanding of underlying disease mechanisms.

## Introduction

Complications involving the central nervous system (CNS) occur in 9–14% of patients following allogeneic hematopoietic stem cell transplantation (HSCT) (1). These include drug toxicities, infections, and metabolic disturbances (2, 3). Graft versus host disease (GvHD) associated with allogeneic HSCT usually affects skin, gut, and liver. GvHD rarely affects the CNS, but when it is involved there is often a significant systemic GvHD elsewhere (4). Patients with chronic GvHD affecting the CNS may present with stroke-like episodes, transverse myelitis, multiple sclerosis (MS) or acute disseminated encephalomyelitis (ADEM) like disorders, encephalitis, and other nonspecific neurological symptoms (5).

Secondary autoimmune diseases, particularly thyroid and other endocrine disorders are recognized complications following allogeneic HSCT, but MS-like presentation has rarely been reported in the literature (5–8). In some cases, there is apparent adoptive transfer of specific autoimmune diseases or autoimmune diathesis (9). Here we report a case of “MS-like” relapsing remitting encephalomyelitis following allogeneic HSCT, which did not respond to three disease modifying therapies (DMTs) and “domino” autologous HSCT.

## Case Presentation

A 53-year-old male with chronic myeloid leukemia (CML), who failed to respond to imatinib (tyrosine kinase inhibitor), underwent allogeneic HSCT for lymphoid blast transformation eighteen months after his initial presentation. Following lymphoid blast transformation, imatinib was also switched to nilotinib (tyrosine kinase inhibitor). The allogeneic HSCT was performed using a conditioning regimen consisting of fludarabine, busulphan and anti-thymocyte globulin (ATG) with ciclosporin A as GvHD prophylaxis. An unrelated male donor matched for HLA A, B, C, and DR loci was used (Supplementary Table 1). Neither donor nor patient had the HLA-DRB1^*^15:01 genotype associated with increased risk of MS (10). Engraftment of neutrophils and platelets occurred promptly, but the patient had routine transplant related toxicities, including post-transplant cytomegalovirus (CMV) reactivation and possible posterior reversible encephalopathy syndrome for which GvHD prophylaxis was switched from ciclosporin A to tacrolimus. There were no changes suggestive of demyelination on brain magnetic resonance imaging (MRI). Tacrolimus was successfully weaned off without GvHD. In view of the high relapse risk, he was maintained on nilotinib post-transplant. Subsequent bone marrow examinations along with peripheral blood monitoring showed full donor chimerism and molecular negativity for BCR-ABL transcripts confirming ongoing molecular remission of leukemia consistent with cure.

**Figure 1:**
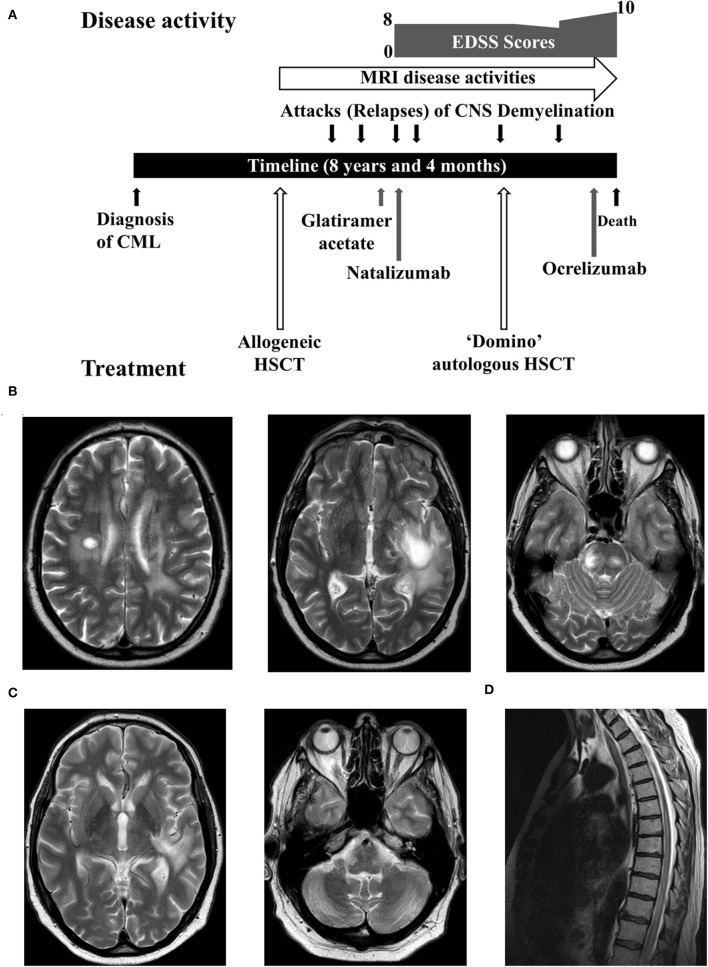
**(A)** A summary of the disease course. **(B)** Axial sections of the brain MRI showed tumefactive lesions. **(C)** Improvement of the edema around tumefactive lesions. **(D)** The spine MRI demonstrated a lesion extending from the thoracic cord to conus.

Despite a good initial recovery, 10 months later the patient was admitted with confusion, slurred speech, left sided facial weakness, and ataxia (Figure 1A). He had no systemic features suggestive of GvHD, such as rash, deranged liver function, or gastrointestinal disturbance. A brain MRI showed contrast enhancing tumefactive lesions in the left peri-insular area, both corona radiatae and brainstem (Figures 1B,C). Screenings for human immunodeficiency virus (HIV), *Borrelia burgdorferi*, syphilis and toxoplasmosis in serum were negative. Analysis of the cerebrospinal fluid (CSF) showed a white cell count of 3 × 10^6^ /L with marginally elevated protein of 0.76 g/L and normal CSF to serum glucose ratio. He had matching bands in CSF and serum (type 4). Screenings for herpes simplex virus (type 1, 2, 6, and 7), varicella zoster virus, adenovirus, enteroviruses, Epstein-Barr virus (EBV), CMV and John Cunningham (JC) virus in CSF were negative. Immunophenotyping of CSF cells and a computed tomography (CT) scan of thorax, abdomen and pelvis did not detect any new or recurrent malignancy. Autoantibody screening for connective tissue diseases and systemic vasculitis were negative.

**Figure 2:**
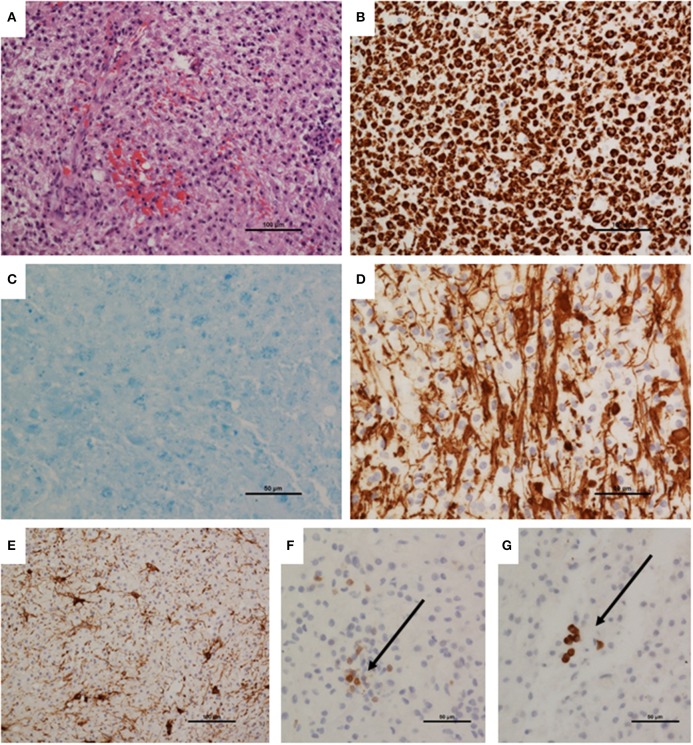
Brain biopsy neuropathology. **(A)** Biopsy appeared hypercellular, with a dense macrophage infiltrate. **(B)** Infiltrating population was confirmed as macrophages by immunohistochemistry to CD68. **(C)** Luxol fast blue stain demonstrating virtually total myelin loss. **(D)** Immunohistochemistry demonstrated relative preservation of axons; these were separated by infiltrating macrophages, and show irregularity, representing damage. **(E)** Immunohistochemistry to GFAP showing reactive astrocytes. **(F)** Immunohistochemistry to CD3 demonstrated sparse T cells (arrow). **(G)** A few CD79a-positive B cells were also present. Magnifications as shown on scale bars.

The patient underwent a brain biopsy, which showed a heavy infiltration of macrophages (CD68-positive), with isolated T (CD3-positive), and B (CD79a-positive) lymphocytes. A luxol fast blue stain revealed severe myelin loss whilst immunohistochemistry to neurofilament protein revealed preserved axons, although some were showing damage and swellings. Reactive astrocytes were present. There was no frank necrosis. Apoptotic cells were not conspicuous and there was no neoplastic infiltration. Special stains for bacteria and fungi were negative, as was immunohistochemistry for JC virus (SV40 antigen). The biopsy findings of a macrophage rich lesion with severe myelin loss but relative axonal preservation suggested a demyelinating pathology (Figure 2). Culture, 16s rRNA gene detection test, screening for pan-fungal and *Aspergillus sp*. were negative in the biopsy sample.

In the absence of systemic features suggesting GvHD, infections and relapse of CML, his clinical presentation and investigation results were considered to be in keeping with possible ADEM. He was treated with a 3-day course of intravenous methylprednisolone followed by tapering doses of oral prednisolone. His neurological symptoms gradually resolved, and he was walking 3−5 miles daily without assistance.

Six months later, he was re-admitted with a 3-week history of paraesthesia and weakness of lower limbs and urinary retention. A brain MRI revealed a new enhancing lesion in the occipital horn adjacent to the left lateral ventricle and a spine MRI showed a contrast enhancing lesion in the thoracic cord extending to the conus (Figure 1D). Similar to previous lumbar puncture results, CSF analysis showed mildly elevated protein with matching bands in CSF and serum. Neuromyelitis optica (NMO) antibody in serum was negative. He was treated with another course of intravenous methylprednisolone and a tapering dose of oral prednisolone. He made a full recovery within several weeks and continued to walk 3–5 miles a day. An enquiry to the donor registry confirmed that the donor remained fit and healthy without neurological or autoimmune diseases.

This case therefore posed a unique diagnostic challenge of differentiating between CNS demyelinating disorders and “pure” CNS GvHD. This presentation neither fulfilled the international consensus diagnostic criteria for NMO spectrum disorder nor the Grauer et al. criteria for the CNS manifestations of GvHD due to the lack of systemic involvement (4, 11). The relapsing remitting disease course was unlikely to be caused by fludarabine or nilotinib toxicity. As the clinical and radiological presentations together with brain biopsy results were thought to be more compatible with relapsing remitting MS, he was commenced on glatiramer acetate and continued on oral prednisolone 5 mg daily.

Two months later, he had another episode of severe myelitis, which was treated with a further course of intravenous methylprednisolone. As the CSF was negative for JC virus, glatiramer acetate was switched to natalizumab, but his disability did not improve and his expanded disability status scale (EDSS) score remained 8.0.

The patient continued to deteriorate requiring another admission with left sided facial and arm weakness five months after starting natalizumab. In view of the diagnostic uncertainty the patient was continued to be investigated for an alternative diagnosis. A spine MRI showed an enhancing lesion in the cervical cord. A third lumbar puncture did not show any new changes and JC virus in CSF remained negative. He was treated with another course of intravenous methylprednisolone. Over the ensuing 15 months, serial MRI scans identified multiple new enhancing lesions in the brain and spinal cord. Visual evoked potentials did not show any evidence of optic nerve involvement on two separate occasions. NMO and myelin oligodendrocyte glycoprotein (MOG) antibodies in serum remained negative.

As he had highly active disease clinically and radiologically despite treatment with a high efficacy DMT, autologous HSCT was considered. As his hematopoietic system was entirely derived from the matched unrelated donor used for the allogeneic HSCT, this was a “domino” autologous HSCT where the patient, a recipient of the previous allogeneic transplant, served as a “donor” for his second transplant. Natalizumab was discontinued and his peripheral blood hematopoietic stem cells were mobilized with cyclophosphamide 2 g/m^2^ and granulocyte-colony stimulating factor. This admission was complicated by confusion secondary to hyponatremia and a urinary tract infection from which he fully recovered.

A cautious approach was adopted, with a decision to observe the neurological response following cyclophosphamide-primed stem cells mobilization, before pursuing with the “domino” autologous HSCT. However, the patient was re-admitted with dysarthria and dysphagia three months later and a brain MRI showed four new enhancing lesions. A decision was therefore made to proceed with the “domino” autologous HSCT following immunoablation with cyclophosphamide and ATG. Similar to the first transplant, the engraftment of neutrophils and platelets occurred promptly. He had routine transplant related toxicities and also made an unhindered recovery following discharge from hospital. Nilotinib was discontinued after the transplant. Three months later his EDSS score was 7.0.

Twelve months after the transplant, he was admitted with confusion, swallowing difficulties and aspiration pneumonia. A brain MRI revealed new enhancing lesions in the left occipital lobe. He was re-investigated for opportunistic infections and malignancies. Sputum culture, throat swabs, galactomannan, and β-d-glucan tests, multiple blood cultures and viral screenings for HIV, hepatitis B and C, syphilis, toxoplasmosis and cryptococcus were negative. Autoimmune screening for connective tissue disease and vasculitis were also negative. A lumbar puncture showed CSF protein of 0.99 g/L and white cell count of 56 × 10^6^ /L with lymphocytosis. CSF to serum glucose ratio was normal. CSF culture and screening for JC virus, EBV, CMV, toxoplasmosis, Cryptococcus, and acid-fast bacilli were negative. On this occasion, there were matching bands in serum and CSF as well as additional monoclonal bands. Neither immunophenotyping of cells in CSF nor CT scan of chest, abdomen and pelvis showed any evidence of recurrence or new malignancy. A second brain biopsy was offered but declined by the patient.

Over the next ten months, he had several brain and spine MRIs, which continued to show radiologically active disease with new T2 lesions and contrast enhancements. He continued oral prednisolone 5 mg daily. He was started on ocrelizumab but died of progressive disease four months later.

**Figure 3:**
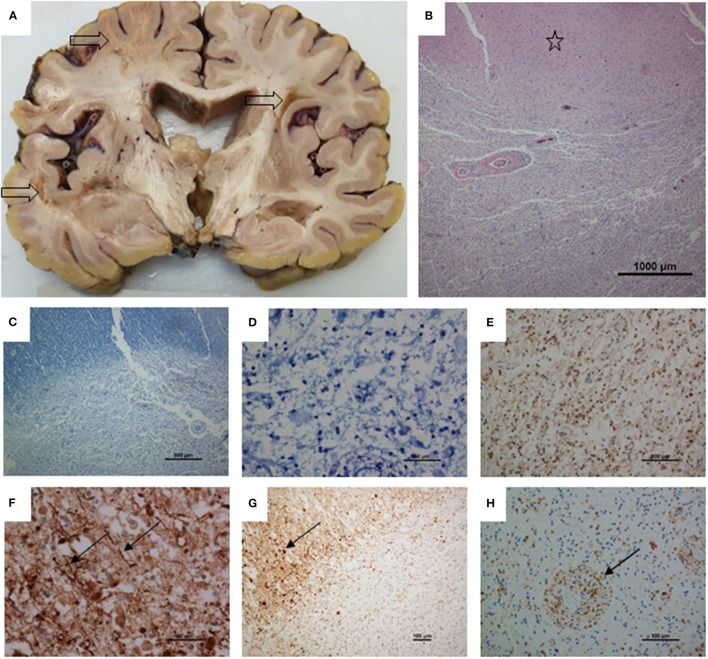
Post-mortem neuropathology. **(A)** Coronal slice of cerebrum showing three irregular, cavitating white matter lesions (arrows). There is some dilatation ex vacuo of the ventricles, presumably secondary to white matter loss. **(B)** H and E stained section showing part of a lesion with preserved cortex above (star). **(C)** Luxol fast blue showing loss of myelin. **(D)** High power view of lesion stained with luxol fast blue shows total loss of myelin. **(E)** Neurofilament immunohistochemistry showing total axon loss in the center of a lesion. **(F)** Toward the lesion margin a few axons remain (arrows). **(G)** Up-regulation of amyloid precursor protein, upper left, with multiple axonal spheroids (arrow). **(H)** CD68 staining showing ameboid microglia, particularly in a perivascular location. Magnifications as shown on scale bars.

The patient underwent a post-mortem limited to the examination of the brain which weighed 1,208 g and was examined after fixation. Coronal slices of the brain revealed multifocal, irregular white matter lesions in frontal, temporal, and occipital lobes, measuring up to 30 mm in diameter. The lesions had a yellowish granular appearance with a tendency to cavitation. Subcortical arcuate (U-) fibers were not spared. These lesions did not have the appearance of classical MS plaques. In places lesions were sharply circumscribed but elsewhere had more diffuse margins. Myelin stains demonstrated virtually total myelin loss and neurofilament immunohistochemistry showed severe axonal loss with a few remained axonal threads at the lesion margins in contrast to the biopsy. Beta amyloid precursor proteins was upregulated in axons and pyramidal cells at the lesion margins. Ameboid macrophages were highlighted by CD68, particularly in a perivascular location but immunohistochemistry to CD3 and CD20 did not reveal T or B cell infiltration. Occasional vessels showed perivascular sclerosis, but immunohistochemistry to smooth muscle actin showed preservation of vascular media suggesting that there was not a vasculopathy process. S100 labeled small round nuclei in the lesions, suggesting some preservation of oligodendrocytes. No oligodendroglial inclusions were seen and immunohistochemistry for JC virus (SV40 antigen) was negative, as were stains for bacteria and fungi. Focal brown pigment was present, staining with both the Perl's and Masson Fontana methods, suggesting that haemosiderin and some melanin was present. The findings were of a multifocal leukoencephalopathy, with severe loss of both axons and myelin (Figure 3).

## Discussion

This is an unusual case of MS-like relapsing remitting encephalomyelitis following allogeneic HSCT, which did not respond to DMTs and “domino” autologous HSCT. This patient's disease course run an initial relapsing remitting phase with complete neurological recovery which was followed by a progressive disease phase with superimposed acute episodic neurological dysfunction. There were corresponding MRI disease activities in brain and spine throughout the disease course.

The initial brain biopsy showed a macrophage rich lesion with loss of myelin, but relative preservation of axons, which would be consistent with a demyelinating pathology. It is a rare histological feature of hematological neoplasm and may be erroneously diagnosed as inflammatory demyelination if corticosteroid therapy is used prior to the brain biopsy (20). Acute plaques of demyelination usually have T cells, which tend to be localized perivascularly and are less prevalent than macrophages (21). In tumefactive MS, T cells are detected at lower levels than in biopsies that subsequently turn out to be lymphoma (20). However in a small biopsy sample, it is possible that these changes may not be well represented, which is a ubiquitous problem independent of disease studied (21). In the absence of clinical features suggestive of GvHD and CML recurrence, his brain biopsy was thought to be consistent with a primary inflammatory demyelinating plaque.

The use of fludarabine has previously been associated with monophasic diffuse necrotizing leukoencephalopathy (22). Although five cases of CNS demyelination had also been reported in patients receiving treatment with nilotinib or imatinib for various malignancies, it was not clear if tyrosine kinase inhibitors were the causal agents, particularly as two out of those five patients recovered without discontinuing these drugs (23–25). Furthermore, tyrosine kinase inhibitor had therapeutic benefit in people with progressive forms of MS and drug toxicities are unlikely to present with a relapsing remitting disease course (26).

There was further discordance between the clinical presentation and the autopsy findings, which showed a destructive leukoencephalopathy. Although the initial biopsy showed demyelination, lymphocytic infiltration was not a feature of either the biopsy or the white matter lesions at autopsy. It is not known how DMTs and HSCT modulate the histological appearance of CNS demyelination. Although the innate immune system recovers within weeks after HSCT, the reconstitution of adoptive immune system occurs over several years. Immunohistochemistry to CD3 and CD20 did not show any T or B cell infiltration during autopsy examination. In particular, ATG was used for *in vivo* T cell purging during domino autologous HSCT. Furthermore, the patient received two doses of ocrelizumab prior to his death. This humanized anti-CD20 monoclonal antibody targets B lymphocytes. The lack of inflammatory cells in the autopsy histology samples could be related to primary underlying pathology or the effect of ocrelizumab and / or the HSCT received earlier.

To our knowledge this was the first patient with MS-like neuroinflammation following allogeneic HSCT, who was treated with a “domino” autologous HSCT. Our patient experienced an aggressive disease course and rapidly became disabled. His failure to respond to glatiramer acetate and natalizumab left his neurologists with limited treatment options. Although the use of alemtuzumab was not completely contraindicated, caution was exercised, as it could cause a prolonged period of lymphopenia potentially making it a less appropriate choice given his immunosuppressed state following allogeneic HSCT (27). Autologous HSCT has been increasingly used to treat patients with MS, who have highly active disease clinically and radiologically, as the safety and efficacy of this procedure has increased over the years through improvement of patient selection, optimization of transplant technique and increased center experience (28). This was therefore thought to be the best treatment option. Although the procedure was associated with routine and well-tolerated toxicities, the response was only transient and failed to achieve long-term remission. In this case, we chose a clinical decision pathway directed at MS, with the use of three DMTs and HSCT, whereas the management of chronic GvHD would have been significantly different. Calcineurin inhibitors, higher doses of steroids, mycophenolate and even extracorporeal photopheresis could have been used for GvHD. We can only speculate whether GvHD management would have made a greater impact on the course of his CNS inflammation compared with a DMT-based, MS-directed approach, even though systemic GvHD was not present.

Reported cases of CNS demyelinating disorders following allogeneic HSCT are very limited. Tables 1, 2 summarize 20 such cases that have been reported in the literature (5–8, 12–19). The median age of receiving allogeneic HSCT was 45.5 (range, 17–65) years and the median interval between HSCT and the onset of CNS demyelination was 1 (range, 0.1–8) year. Twelve of these patients presented with neurological symptoms within 1 year of allogeneic HSCT and remaining eight patients developed neurological symptoms after 2 years or more. Male to female ratio was 3: 1. There was evidence of GvHD in 12 patients and peripheral nerves involvement was reported in 13 patients. Inflammation less frequently affected brainstem, cerebellum and meninges. CSF analysis was normal in only 6 patients and oligoclonal bands were present in 7 patients.

**Table 1:** Demographic details, allogeneic HSCT procedures, GvHD and other immune mediated complications of post-transplant CNS demyelinating disorders.

**No**	**Age of HSCT**	**Gender**	**Initial disease**	**Donor**	**Conditioning regimen**	**GvHD prophylaxis**	**GvHD history**	**Peripheral nerve involvement**	**References**
1	24	Male	Lymphoblastic T cells lymphoma	HLA (B, C DR identical and A mismatched) mixed lymphocytic culture non-reactive mother	NA	NA	Liver and cutaneous	Yes	(12)
2	32	Female	MDS	Matched related donor	Bu and Cy	CsA and Methotrexate	Liver and Cutaneous^*^	NA	(13)
3	58	Male	CMML	Unrelated donor	Bu and Flu	ATG, Methotrexate, and Tacrolimus	No	NA	(6)
4	65	Male	AML	Matched related donor	TBI, Cy Flu, Amsacrine, Cytarabine, and ATG	CsA and MMF	No	NA	(6)
5	50	Male	Myeloproliferative neoplasms	Matched related donor	Myeloablative regimen	CsA and MMF	Muscle	NA	(5)
6	36	Male	ALL	Matched related donor	NA	CsA and Tacrolimus	Gut	NA	(7)
7	35	Female	MDS	Matched unrelated donor	Bu, Flu, and ATG	Methotrexate	Cutaneous and liver	NA	(14)
8	41	Male	Aplastic anemia	Matched unrelated donor	TBI, Cy, and ATG	CsA and Corticosteroids	Lung, muscle, and cutaneous^*^	NA	(15)
9	59	Male	AML	Matched related donor	TBI, Cy, Flu, Cytarabine, and GCSF	ATG, CsA, and MMF	Cutaneous	Yes	(8)
10	55	Male	AML	Matched unrelated donor	NA	NA	No	Yes	(8)
11	53	Male	AML	Matched related donor	TBI, Cy, Flu, Cytarabine, and Amsacrine	ATG, MMF, and Tacrolimus	No	Yes	(8)
12	56	Male	CMML	Matched unrelated donor	Bu and Flu	ATG, Methotrexate, and Tacrolimus	No	Yes	(8)
13	53	Female	CML	Matched related donor	NA	NA	No	Yes	(8)
14	33	Male	Hodgkin lymphoma	Matched unrelated donor	Flu and Melphalan	ATG, MMF, and Tacrolimus	Cutaneous and muscle	Yes	(8)
15	54	Male	AML	Matched related donor	NA	NA	No	NA	(16)
16	59	Male	AML	Matched related donor	NA	NA	No	NA	(16)
17	29	Female	AML	Matched related donor	NA	NA	Cutaneous	NA	(16)
18	40	Male	CML blast crisis	NA	Bu and Cy	CsA and Corticosteroids	Cutaneous	NA	(17)
19	36	Female	MDS	Matched unrelated donor	Bu and Cy	CsA and methotrexate	Possible cutaneous^*^	NA	(18)
20	17	Male	AML	Matched related donor	NA	CsA and Corticosteroids	No	NA	(19)

* *Patient also had cytopenia*.

*ALL, Acute lymphoblastic leukemia; AML, Acute myeloid leukemia; ATG, Anti-thymocyte globulin; Bu, Busulfan; CML, Chronic myeloid leukemia; CMML, Chronic myelomonocytic leukemia; Cy, Cyclophosphamide; Cyclosporin-A, CsA; Flu, Fludarabine; GCSF, Granulocyte colony stimulating factor; HSCT, Hematopoietic stem cell transplantation; GvHD, Graft versus host disease; MDS, Myelodysplastic syndrome; MMF, Mycophenolate mofetil; NA, Not available; and TBI, Total body irradiation*.

**Table 2:** Clinical features, investigation results and treatment outcomes of post-transplant CNS demyelinating disorders.

**No**	**Clinical syndrome**	**HSCT to onset (year)**	**Location of MRI lesions**	**CSF**	**Biopsy**	**Clinical course**	**Follow up period (year)**	**Treatment**	**Treatment response**	**Residual disabilities**
1	MS-like	1	Brainstem, cerebellum, and corona radiata	OCB (Type 4) and lymphocytosis	No	Relapsing remitting	2.3	Corticosteroids and TPE	Marginal improvement	Wheelchair bound
2	ON with myelitis	0.6	Internal capsule, thalamus, and cervical cord with Gd- enhancement	Normal	No	Relapsing remitting	1	Corticosteroids and Cs A	Partial improvement	Had residual deficits
3	Myelitis	0.1	Cervical and thoracic cord	Raised protein	No	Relapsing remitting	4.5	Corticosteroids, CsA, and Cy	Partial improvement	Was able to walk 500 m
4	MS-like	3	Left hemisphere, cervical, and thoracic spine with Gd-enhancement	Raised protein and OCB	No	Relapsing remitting	3	Corticosteroids	Complete resolution	Nil
5	MS-like	7	Compatible with MS	Normal	No	NA	8.3	Cs A	Complete resolution	Nil
6^**^	MS	2	Corpus callosum, right temporal subcortex, cervical, and thoracic cord with Gd-enhancement	Normal	Brain biopsy—complete loss of myelin compatible with MS	Relapsing remitting	3	Corticosteroids and Interferon β	Complete resolution	Nil
7^**^	ADEM	2	Fontal subcortex, periventricular area, occipital lobe, and thoracic cord	Normal	Brain biopsy—loss of myelin thought to be GvHD and spine biopsy showed fibrosis	Single episode	1	Corticosteroids, TPE, and Tacrolimus	Complete resolution	Nil
8	Myelitis	0.5	Cervical and thoracic cord	Raised protein	No	One episode and a possible relapse	3	Corticosteroids	Complete resolution	Nil
9^**^	ON with myelitis	8	Bilateral pre- and post-chiasm, cervical, and thoracic cord and meningeal enhancement, cervical	Pleocytosis with OCB (type 2)	No	NA	NA	Corticosteroids, IVIG, Rituximab	Marginal improvement	Unable to stand up or walk
10^**^	MS	2.6	Brainstem and periventricular lesions	Raised protein with OCB (type 2)	NA	NA	NA	Corticosteroids, Interferon β, Rituximab	Marginal improvement	Spastic quadriparesis and gait instability
11^**^	NMOSD	1	Left pre-chiasmatic lesions and LETM in thoracic and lumbar cord	Raised protein, and pleocytosis	No	NA	NA	Corticosteroids, TPE, Rituximab	Marginal improvement	Visual impairment and spastic paraparesis
12	Myelitis	0.25	Cervical and thoracic spine	NA	No	NA	NA	Corticosteroids and Cy	Marginal improvement	Spastic quadriparesis and gait Instability
13^**^	Myelitis	5	Spinal cord WM and cerebral peduncles	Normal	No	NA	NA	Corticosteroids	No improvement	Left sided spastic hemiparesis, gait instability, and sensory deficits
14^**^	ON with myelitis	0.33	Periventricular lesions, optic nerve atrophy, and spinal cord WM	Raised protein, and pleocytosis	Sural nerve biopsy—demyelination and axonal degeneration	NA	NA	Corticosteroids, TPE, IVIG, and Rituximab	Marginal improvement	Spastic quadriparesis, visual impairment, and gait instability
15	ADEM	1	Subcortical enhancing lesions	Raised protein and pleocytosis	Brain biopsy—loss of myelin	Single episode	5	Corticosteroids, and IVIG	NA	Mild residual deficit, but died from second malignancy
16	ADEM	0.66	Brainstem	Raised protein and OCB	No	Single episode	9	IVIG	NA	Mild deficit and still alive
17	ADEM	0.17	Brain and cervical cord	Raised protein and OCB	Spine biopsy—loss of myelin	Single episode	2	Corticosteroids, IVIG, and donor lymphocyte infusion	Marginal improvement	Sever deficit, bed bound, and died for severe GvHD induced generalized scleroderma
18	Myelitis	2.25	Cervical and thoracic cord with enhancement	OCB	No	Relapsing remitting	1.25	Corticosteroids and TPE	Partial improvement	Ambulatory with a cane
19	Myelitis	0.25	Multifocal enhancing, lesion in cervical, and thoracic spine	Normal	No	Relapsing remitting	1.5	Corticosteroids	Significant improvement	Nil significant
20	NMOSD	0.15	Brain WM, cervical, and thoracic cord	Pleocytosis	No	Relapsing remitting	1.5	Corticosteroids TPE, IVIG, and MMF	Partial improvement	Visual acuity and sensory deficit

** *NMO antibody was examined and it was negative. ADEM, Acute disseminated encephalomyelitis; CSF, Cerebrospinal fluid; Cy, Cyclophosphamide; Cyclosporin-A, CsA; Gd, Gadolinium; HSCT, Hematopoietic stem cell transplantation; IVIG, Intravenous immunoglobulin; LETM, Longitudinally extensive transverse myelitis; MRI, Magnetic resonance imaging; MS, Multiple sclerosis; MMF, Mycophenolate mofetil; NA, Not available; NMOSD, Neuromyelitis optica spectrum disorder; OCB, Oligoclonal band; ON, Optic neuritis; TPE, Therapeutic plasma exchange and WM, White matter*.

The clinical course of these patients was monophasic or relapsing remitting. They had variable clinical presentations including optic neuritis with myelitis, pure myelitis, ADEM, and MS-like neuroinflammation (Table 2). The diagnostic criteria for MS and NMO spectrum disorder were satisfied in two patients each. NMO antibody was absent in all cases where it was examined (Table 2). Terminologies such as “central and peripheral nervous system immune-mediated demyelinating disease (CPID)” and “immune-mediated demyelinating disease (IMDD)” had been coined by some authors to refer these presentations as a rare late onset complication of allogeneic HSCT (8, 16). Some authors also suggested that these presentations may be CNS manifestation of GvHD (5). Three patients had brain biopsy, two had spine biopsy and another person had sural nerve biopsy. All of these biopsies showed loss of myelin and the brain biopsy of one patient was compatible with GvHD (Table 2). None of those case reports included post-mortem examination.

One possible pathophysiological mechanism of CNS demyelination following allogeneic HSCT could be immune mediated damage due to minor histocompatibility differences between brain tissues and the graft derived cells. Another possible mechanism is adoptive transfer of autoimmunity resulting in CNS inflammation in the host.

Complete resolution of symptoms or significant improvement was observed in 6 patients and partial improvement was reported in another 4 patients (5–7, 14, 18, 19). Eight of them had marginal or no improvement following treatment and data were not available for 2 patients (5–8, 13, 16). A range of therapies including corticosteroids, intravenous immunoglobulin, therapeutic plasma exchange, mycophenolate, tacrolimus, cyclosporine A, cyclophosphamide, rituximab, and donor lymphocyte infusion had been used in these 20 patients (Table 2). Interferon ß was used in both patients, whose diagnoses were compatible with MS. Five patients, who were treated with mycophenolate, tacrolimus or cyclosporine A, had complete resolution of symptoms or partial improvement following treatment. The size of the sample was too small for any statistical analysis, but normal CSF constitutes or absence of oligoclonal bands were appeared to be associated with better prognosis. Complete resolution of symptoms or significant clinical improvement was observed more frequently in those patients who had later onset of neurological symptoms following allogeneic HSCT (Table 2).

## Conclusions

This case was noteworthy for two reasons. Firstly, the disease course which resembled an “MS-like” relapsing remitting encephalomyelitis although the exact etiology remained unknown even after autopsy. In such a case full autopsy examination may be helpful to confirm the absence of occult malignancy and also to investigate whether the disease was limited to the CNS tissue. Secondly, this case demonstrated the feasibility of using “domino” autologous HSCT in patients presenting with “MS-like” encephalomyelitis following allogeneic HSCT, but further studies are required to evaluate its safety and efficacy.

## Ethics Statement

A written consent was obtained from the relative of the patient for the publication of this case report.

## Author Contributions

JD summarized the case, reviewed the literature and drafted the manuscript. SP, SW, BS, and JS drafted the manuscript, AG, CL, NC-L, and HJ reviewed and summarized the case.

## Conflict of Interest

JS declares speaker fees at educational events supported by Sanofi, Janssen, Jazz, Mallinckrodt and Gilead. JS is a member of a trial IDMC for Kiadis Pharma. The remaining authors declare that the research was conducted in the absence of any commercial or financial relationships that could be construed as a potential conflict of interest.
