# Supplementary material for: A Case of Multiple Sclerosis—Like Relapsing Remitting Encephalomyelitis Following Allogeneic Hematopoietic Stem Cell Transplantation and a Review of the Published Literature
Source: Front Immunol. 2020 May 5;11:668. doi: 10.3389/fimmu.2020.00668 (PMC7214636; doi:10.3389/fimmu.2020.00668)
Supplement: Supplementary file 1 [file Table_1.DOCX]

**Supplementary Table 1:** Summary of the histocompatibility and immunogenetics between the patient and the donor

| **HLA** | **A^*^** | | **B^*^** | | **C^*^** | | **DRB1^*^** | | **DQB1^*^** | | **DPB1^*^** | |
| --- | --- | --- | --- | --- | --- | --- | --- | --- | --- | --- | --- | --- |
| **Patient** | 02:01 | 03:01 | 07:02 | 55:01 | 03:03 | 07:02 | 01:03 | 15:01 | 06:02 | 03:01 | 01:01 | 04:02 |
| **Donor** | 02:01 | 03:01 | 07:02 | 55:01 | 03:03 | 07:02 | 01:03 | 15:01 | 05:01 | 06:02 | 03:01 | 05:01 |
